# Costs of a Brief Alcohol Consumption Reduction Intervention for Persons Living with HIV in Southwestern Uganda: Comparisons of Live Versus Automated Cell Phone-Based Booster Components

**DOI:** 10.1007/s10461-023-04010-6

**Published:** 2023-02-20

**Authors:** Judith A. Hahn, Sebastian Kevany, Nneka I. Emenyonu, Naomi Sanyu, Anita Katusiime, Winnie R. Muyindike, Robin Fatch, Starley B. Shade

**Affiliations:** 1 University of California, 550 16th Street, 3rd Floor, San Francisco, 94158 USA; 2 Asia-Pacific Center for Security Studies, Hawaii, USA; 3 Mbarara University of Science and Technology, Mbarara, Uganda; 4 United Nations University, Tokyo, Japan; 5 University of Leeds, Leeds, United Kingdom

**Keywords:** Alcohol, Costing, HIV, Brief intervention, Sub-Saharan Africa, Mobile health, Technology

## Abstract

Low-cost interventions are needed to reduce alcohol use among persons with HIV (PWH) in low-income settings. Brief alcohol interventions hold promise, and technology may efficiently deliver brief intervention components with high frequency. We conducted a costing study of the components of a randomized trial that compared a counselling-based intervention with two in-person one-on-one sessions supplemented by booster sessions to reinforce the intervention among PWH with unhealthy alcohol use in southwest Uganda. Booster sessions were delivered twice weekly by two-way short message service (SMS) or Interactive Voice Response (IVR), i.e. via technology, or approximately monthly via live calls from counsellors. We found no significant intervention effects compared to the control, however the cost of the types of booster sessions differed. Start up and recurring costs for the technology-delivered booster sessions were 2.5 to 3 times the cost per participant of the live-call delivered booster intervention for 1000 participants. These results suggest technology-based interventions for PWH are unlikely to be lower cost than person-delivered interventions unless they are at very large scale.

## Introduction

Alcohol use is a key driver of the HIV pandemic, fuelling reduced medication adherence, sexual risk behavior, and long-term health outcomes [1–3]. A substantial number of new HIV infections in sub-Saharan Africa (SSA) are attributable to unhealthy alcohol use (i.e. drinking above safe limits [4]); one modelling study based on an intervention trial conducted in Kenya suggested that approximately one-third of new alcohol-related HIV infections could be averted by an alcohol reduction intervention with high efficacy [5]. Data on the efficacy of behavioral interventions to reduce alcohol use among people with HIV (PWH) have ranged from inconclusive [6] to positive [7], and the World Health Organization has called for interventions to reduce alcohol use for hazardous drinkers worldwide [8].

Recently, there has been substantial interest in interventions that can leverage remote delivery of interventions via cell phones to improve the health of PWH [9, 10], including interventions that target substance use [11, 12]. A meta-analysis found that personalized digital interventions, delivered by computers, mobile devices, and smartphones reduced alcohol use in the general population by an average of 3 drinks per week (which would be a small reduction for those with alcohol use disorder[13]) compared to those in the control conditions, which included no or minimal interventions [14]. An advantage of cell phone-based interventions is that they may reduce costs compared to face-to-face sessions, given their ability to be automated and scaled up more easily than in-person-based interventions. This may be especially relevant for SSA which has experienced dramatic increases in cell phone use [15]. Automated cell-phone based interventions have the potential to increase the frequency of delivery, and may therefore be very important for counseling-based alcohol interventions, in which repeated contact, rather than duration, was shown to be a key factor in their success [16].

Three studies have reported on the cost-effectiveness of alcohol interventions in low- and middle-income countries (LMICs). These include a study of two interventions to reduce alcohol use (a combined intervention of cognitive behavioral therapy with motivational enhancement therapy and a brief counselling-based intervention, similar to the one described below) delivered to persons with HIV in Vietnam [17] that reported costs of $95 and $39 per participant receiving the combined and brief interventions, respectively [18]; these were found to be cost-effective compared to standard of care [19]. Cost-effectiveness was demonstrated for a brief intervention targeted to men with heavy alcohol use in Goa, India, assuming $35 per participant [20], and a simulation study found that several targeting scenarios were cost-effective for a hypothetical very low-cost ($5 per person) very brief intervention for PWH in Kenya [21]. Taken together, these studies suggest that low-cost efficacious interventions are likely to be cost-effective. However, we are not aware of any studies that have examined the cost of incorporating cell-phone delivered intervention components to reduce alcohol use in PWH in LMICs.

Because of the deleterious role that alcohol use plays on the HIV epidemic in low-resource settings, we undertook a study to examine a brief counselling intervention found to be efficacious for reducing alcohol use [17, 22] and increasing viral suppression [17] in PWH elsewhere. Through a series of focus groups and individual interviews, we adapted this counselling-based intervention to the local context [23] and included technology to deliver parts of the intervention. The study aimed to examine intervention efficacy, feasibility, acceptability, and cost; here, we report on the costs. The study was a three-arm randomized controlled trial of two versions of a brief counselling-based intervention, each compared to a control arm. The intervention was comprised of two in-person sessions three months apart, plus booster sessions in between the in-person sessions to reinforce the counselling. We randomized participants to receive the intervention with the booster calls delivered approximately monthly by phone by live counsellors (live call arm), or delivered twice weekly using two-way automated systems, either interactive voice response (IVR) or Short Message Service (SMS), i.e., text message, as desired by the participant (technology arm). The technology-based intervention components were considered as potentially efficient modes of implementation of interventions to reduce alcohol use among PWH. The main efficacy outcomes of the study were self-reported number of days drinking, the alcohol biomarker phosphatidylethanol (PEth), and HIV viral suppression (< 40 copies/ml). We found high acceptability and feasibility of the intervention, and significant effects of each intervention arm compared to the control of self-reported number of drinking days, but no effects on PEth on viral suppression [24]. Despite the lack of efficacy, we sought to determine the costs of these booster delivery methods, overall and in comparison to methods using less technology, so that these costs may inform future interventions. Therefore, the main goal of this manuscript is to compare costs for the delivery of a brief alcohol intervention by mode of booster session delivery.

## Methods

### Setting and Population

This study was conducted at the Immune Suppression Syndrome (ISS) Clinic of Mbarara Regional Referral Hospital in Southwest Uganda, which is embedded within the second largest medical institution in the country, Mbarara University of Science and Technology (MUST). There are over 11,000 active patients at the Mbarara ISS Clinic. The adult HIV prevalence in Southwest Uganda is 6.3% [25] and heavy alcohol use is common, with over 20% of the adult population consuming more than 4 drinks in one sitting over the past 30 days [26].

The costing was conducted as part of a study called the Extend study (NCT03928418), which included conducting a RCT to examine the efficacy, cost, and acceptability of the intervention. The study procedures were approved by the Institutional Review Boards of the Mbarara University of Science and Technology and the University of California, San Francisco and the Uganda National Council for Science and Technology.

### Extend Study Population and Trial Design

The study eligibility criteria were being a patient at the Immune Suppression Syndrome Clinic of the Mbarara Regional Referral Hospital, on anti-retroviral therapy for at least six months, and having an Alcohol Use Disorders Identification Test – Consumption, modified to represent the prior 3 months, score unhealthy drinking (≥ 3 for women or ≥ 4 (men), and having daily access to a working cell phone. 994 persons were screened, 321 were eligible and 270 were enrolled in the study [24]. They were randomized to (1) in-person brief manualized alcohol counseling at two regularly-scheduled quarterly clinic visits plus interim boosters delivered monthly by phone by a counselor (live call arm); (2)in-person brief manualized alcohol counseling at two regularly-scheduled quarterly clinic visits plus interim boosters delivered twice weekly either by short message service (SMS) or interactive voice response (IVR) (technology arm); or (3) standard of care (SOC) which included brief unstructured advice from clinic staff, with a wait-listed intervention (control arm). Ninety persons were randomized to each arm, but one person was inadvertently randomized twice (to the control arm). The intervention was based on a brief intervention that showed efficacy for reducing self-reported alcohol use among PWH in Baltimore, MD, USA [22] and Thai Nguyen, Vietnam [17], and was adapted for the local context through a series of focus groups and individual interviews [23]. The live call and technology booster sessions were designed to check progress on meeting the drinking agreement made at the first in-person counseling session, and to provide positive reinforcement when drinking was reduced and encouragement when goals were not met. The automated technology booster sessions were delivered using two-way SMS or IVR, based on the participants’ choice; both allowed for brief interactive sessions tailored to the participants’ drinking goals and gender. During these sessions, the participant replied to questions/prompts using the buttons on their phone. The IVR option, which uses voice recordings, was included to account for the expected low literacy of some participants. The technology for the automated calls was developed and tested as part of the formative phases of this study [23] in collaboration with a local technology company (Innovation Streams Limited, istreams); the program is available upon request. All booster sessions were scheduled to occur at the participants’ preferred days and times of choice (approximately every 3 weeks for the live call arm, 2 times per week for the technology arms).

At baseline, the mean number of drinking days of the prior 21 was 9.4 (95% Confidence Interval [CI]: 9.1–9.8), mean PEth was 407.8 ng/mL (95% CI: 340.7-474.8), and 89.2% were virally suppressed [24]. At follow up (6 and 9 months), there were significant reductions in number of drinking days in the live call arm and the technology arm compared to the controls ((3.5, 95% CI:2.1–4.9) and (3.6, 95% CI: 2.2–5.1) respectively). However, there were no significant differences in mean PEth (36.4 ng/mL (95% CI: -117.5-190.3) for the live call arm compared to the controls and − 30.9 ng/mL (95% CI: -194.8-132.9) for the technology arm compared to controls) or in viral suppression (-2.3% (95% CI:-9.3-4.7) for the live call arm compared to the controls, and − 0.9% (95% CI:-8.3-6.4) for the technology arm compared to the controls.

Participants in the SOC arm were invited to receive the intervention 9 months after baseline enrolment (wait list control). Those SOC arm participants accepting the intervention were allowed to choose to receive their boosters via live calls from the counselor, or automated calls via SMS or IVR.

### Data Collection

We captured all service delivery costs, regardless of funding source (e.g., research grant, Uganda Ministry of Health [MOH], other), to better understand the costs of implementation by each mode of booster delivery, separating costs for interventions with boosters delivered by live counselors, IVR, and SMS. We collected costing data between November 2019 and March 2021. We used e-mail and phone conversations to elicit costs for office equipment and training from the MUST Grants Office finance staff and local salary costs from the local investigator and study coordinator. We used a tracking log to record hours worked and distribution of effort by the intervention counselors. Counselor effort included time spent preparing for sessions with participants, in-person counseling sessions, booster phone calls, and administrative activities such as scheduling appointments and organizing session notes. We recorded the effort of other intervention staff such as the data manager and study coordinator; we limited this to activities directly related to the intervention and excluded time spent on research activities adapting the intervention and conducting the RCT. We used a standard exchange rate from September 1, 2019, of 1 US dollar to 3690 Ugandan Shillings. All information was then reviewed with study staff and investigators to double-check accuracy.

### Tools

We used standard costing tools for intervention delivery type cost comparisons. These included data collection spreadsheets for the in-person counseling component of the intervention (which was identical across all booster session delivery types) and for the booster sessions (either live call, SMS, or IVR sessions, analyzed separately here) that we adapted from past costing efforts. We also reviewed counselor tracking logs to determine hours worked per task, which helped to further inform division of staff time across study service delivery types. The data collection spreadsheets also divided cost and effort information across time period (start up vs. implementation), resource category (capital vs. recurring), level of costs (i.e., costs specific to the intervention overall, each counselor, or each participant) and intervention booster delivery type (live call, SMS, IVR).

### Time Periods

We captured information on the time period for each cost based on whether it was consumed before (start-up cost) or during (implementation cost) implementation of the intervention. This helped us to determine whether the intervention was proportionately more expensive to set up or to maintain operation.

### Costing Resource Categories

We used standard resource categories, based on an overarching division between capital (fixed) and recurring (variable) costs over a one-year time period (except for per-participant costs, which were calculated over three months, the typical intervention duration). Capital costs included hardware and software investments (e.g., laptop computers and mobile phone and connectivity equipment and cloud storage), costs for developing the SMS and IVR programs with a local technology company, as well as office equipment and initial staff training. Recurring costs were divided into three main categories: staff salaries and benefits; connectivity and technology support (office network); and other field office costs (office consumables; cell-phone air time).

### Stratification of Costs

We divided costs based on whether they were driven by overall implementation (intervention-level costs), the number of counselors employed (counselor-level costs), or the number of participants enrolled in each intervention (participant-level costs). Intervention-level costs included those that did not vary as the program grew (i.e. infrastructure costs, office equipment, supervisory staff salaries and benefits, and software development and information technology (IT) support costs). Counselor-level costs included those related to an individual counselor (i.e., tables, chairs, phones, and audio recording equipment for intervention fidelity assessment). Per-participant costs included counselor staff time (calculated based on participant-minute resource logs); cell phone credit supplied to participants; workbook photocopying; and other participant-level costs.

### Allocation of Shared Costs

We allocated shared costs based on their use for activities related to each intervention arm versus use for other activities, such as research activities related to the RCT, and removed all non-intervention costs. We divided counselor costs based on the relative time required to conduct the in-person counseling sessions and live booster calls. All other costs were divided based on effort allocation across study booster delivery types, in keeping with standard costing practices. We separated total observed costs into start up and implementation costs.

### Cost per Participant

We calculated costs per participant for the intervention including capital and recurring costs at the level of the intervention, counselor and participant, by combining all related costs per booster delivery type (live call, SMS, IVR) over the analysis period. These were then divided by the number of participants who received each booster session delivery type, including those receiving the intervention in the wait-list control arm, to produce per-participant costs for each booster delivery type. We separated per-participant costs into start up and implementation costs.

### Replication Cost Scenarios

We conducted sensitivity analysis to estimate the costs of scale-up (i.e., replication, no start-up costs included) of the intervention with each booster call delivery type to 1000 participants under four hypothetical scenarios: (1) a stand-alone program implemented by an international non-governmental organization (NGO), aid agency, or other charity; i.e., an organization with out of country funding or a private facility; (2) a program integrated into existing programs within an NGO, aid agency, other charity, or private facility; (3) a stand-alone program implemented by the Ugandan Ministry of Health (MOH); (4) and a program integrated into existing programs within the MOH. Stand-alone programs mean that the resources are not shared with other organization functions, while integrated programs can share staff and other resources. In these scenarios, programs implemented by an international NGO, aid agency, other charity, or private facility are characterized by higher costs needed to support office space outside of MOH facilities, higher salaries of international NGO staff or staff employed in other facilities, and costs to purchase what may be deemed as luxury items such as water coolers and fans.

In the stand-alone models, we assume that the program will hire an additional full-time counselor when the number of participants exceed the number who can be served by the number of existing counselors, while in programs that are integrated into existing programs, we assume that the program will only need to pay existing staff for the proportion of time needed to implement the intervention. In the NGO models, we assume that program oversight staff and counselors will be paid at a wage similar to their salary in the observed trial, while in the MOH models, we assume that program oversight staff and counselors will be paid according the MOH salary scales. We also assume that site rental, utilities, and some ‘luxury’ items would not be included in the costs of MOH models.

We present graphs of the estimated overall cost of these hypothetical scenarios broken out by time period (start-up vs. implementation) and type of cost (capital vs. recurring).

### Economies of Scale

We also compared economy of scale across the interventions that included live counselor, SMS and IVR booster delivery methods by estimating costs per participant if each intervention was implemented with 40 to 3200 participants using each of the four hypothetical scenarios. We present a graph for each hypothetical scenario to compare the potential efficiency of each as the size of the program increases intervention under each hypothetical scenario.

## Results

### Participants

272 participants were enrolled in the study (269 for RCT analysis, 3 for piloting procedures). Overall, 91 participants were randomized to the live call arm, 91 were randomized to the technology-delivered booster arm, and 90 were randomized to the SOC wait-list control arm. Within the technology-delivered booster arm, 31 chose booster delivery by SMS and 60 chose booster by IVR. Among the 90 people randomized to the wait list, when offered the intervention, 69 chose live call delivered booster calls, 10 chose technology-delivered booster calls (9 SMS and 1 IVR), 6 declined any intervention, and 5 were lost to follow up. In total, 160 received the intervention with the live call boosters, and 101 received the intervention with automated technology boosters (40 SMS and 61 IVR).

### Observed Costs

In Tables 1 and 2 we present our observed costs overall and per participant, by time period (start-up versus implementation), using the actual number of participants who received each intervention. The intervention with live call-delivered boosters cost $23,083 over 12 months of implementation or $144.27 per enrolled participant (n = 160); the intervention with SMS-delivered boosters cost $12,512 over 12 months of implementation or $312.79 per enrolled participant (n = 40); the intervention with IVR-delivered boosters cost $19,242 over 12 months of implementation or $315.45 per enrolled participant (n = 61). Implementation costs for the live call booster arm were driven by intervention-level recurring costs such as program oversight (management) staff salaries and benefits, and counselor-level costs such as counselor salaries and benefits. Participant-level costs made up a minority of implementation costs for the live call booster arm. The intervention with SMS-delivered boosters included substantial start-up costs associated with software developed for the intervention. Implementation costs for the SMS-delivered booster arm were driven primarily by intervention-level recurring costs including connectivity and program oversight staff salaries and benefits, rather than counselor-level and participant-level costs. The intervention with IVR-delivered boosters had higher start-up costs than the SMS intervention due to the cost of voice recordings and the use of different technology than the SMS intervention. Implementation costs for the IVR-delivered booster arm were driven by intervention-level recurring costs, particularly for connectivity, followed by program oversight staff salaries and benefits, rather than counselor-level and participant-level costs.

**Table 1:** Overall Observed Start-up and Implementation Costs

Category	Live Counselor		SMS		IVR	
	$	%	$	%	$	%
Start-up Costs						
***Fixed Costs***	***$1,763.38***	***7.64%***	***$4,587.66***	***36.67%***	***$7,980.65***	***41.47%***
Hardware	$0.00	0.00%	$61.48	0.49%	$102.47	0.53%
Software	$774.14	3.35%	$4,042.27	32.31%	$7,139.25	37.10%
Training	$245.20	1.06%	$61.20	0.49%	$93.60	0.49%
Office equipment	$744.04	3.22%	$422.70	3.38%	$645.33	3.35%
**Implementation Costs**						
***Intervention-level Recurring Costs***	***$9,788.70***	***42.41%***	***$5,546.24***	***44.33%***	***$7,889.76***	***41.00%***
Staff salaries and benefits	$7,410.01	32.10%	$1,849.48	14.78%	$2,828.62	14.70%
Connectivity	$398.70	1.73%	$3,676.02	29.38%	$5,029.41	26.14%
Office consumables	$1,980.00	8.58%	$20.75	0.17%	$31.73	0.16%
***Counselor-level Costs***	***$9,523.33***	***41.26%***	***$1,621.61***	***12.96%***	***$2,666.05***	***13.86%***
Counselor time	$9,179.40	39.77%	$1,538.21	12.29%	$2,535.98	13.18%
Office equipment	$260.81	1.13%	$65.10	0.52%	$99.56	0.52%
Office consumables	$83.12	0.36%	$18.30	0.15%	$30.51	0.16%
***Participant-level Costs***	***$2,007.51***	***8.70%***	***$756.19***	***6.04%***	***$705.85***	***3.67%***
Printed intervention material	$1,838.40	7.96%	$459.60	3.67%	$700.89	3.64%
Cell phone credit (counselors)	$169.11	0.73%	$3.25	0.03%	$4.96	0.03%
Cell phone credit (participants)	$0.00	0.00%	$293.33	2.34%	$0.00	0.00%
SMS charges	$0.00	0.00%	$0.00	0.00%	$0.00	0.00%
**Total Costs**	**$23,082.92**	***100.00%***	**$12,511.70**	***100.00%***	**$19,242.30**	**100.00%**

**Table 2:** Observed Start-up and Implementation Costs *per Participant*

	Live Counselor		SMS		IVR	
	$	%	$	%	$	%
Start-up Costs						
***Fixed Costs***	***$11.02***	***7.64%***	***$114.69***	***36.67%***	***$130.83***	***41.47%***
Hardware	$0.00	0.00%	$1.5466	0.49%	$1.68	0.53%
Software	$4.84	3.35%	$101.06	32.31%	$117.04	37.10%
Training	$1.53	1.06%	$1.53	0.49%	$1.53	0.49%
Office equipment	$4.65	*3.22%*	$10.57	3.38%	$10.58	3.35%
**Implementation Costs**						
***Intervention-level Recurring Costs***	***$61.18***	***42.41%***	***$138.66***	***44.33%***	***$129.34***	***41.00%***
Staff salaries and benefits	$46.31	32.10%	$46.24	14.78%	$46.37	14.70%
Connectivity	$2.49	1.73%	$91.90	29.38%	$82.45	26.14%
Office consumables	$12.38	8.58%	$0.52	0.17%	$0.52	0.16%
***Counselor-level Costs***	***$59.52***	***41.26%***	***$40.54***	***12.96%***	***$43.71***	***13.86%***
Counselor time	$57.37	39.77%	$38.46	12.29%	$41.57	13.18%
Office equipment	$1.63	1.13%	$1.63	0.52%	$1.63	0.52%
Office consumables	$0.52	0.36%	$0.46	0.15%	$0.50	0.16%
***Participant-level Costs***	***$12.55***	***8.70%***	***$18.90***	***6.04%***	***$11.57***	***3.67%***
Printed intervention material	$11.49	7.96%	$11.49	3.67%	$11.49	3.64%
Cell phone credit (counselors)	$1.06	0.73%	$0.08	0.03%	$0.08	0.03%
Cell phone credit (participants)	$0.00	0.00%	$7.33	2.34%	$0.00	0.00%
SMS charges	$0.00	0.00%	$0.00	0.00%	$0.00	0.00%
**Total Costs**	***$144.27***	***100.00%***	***$312.79***	***100.00%***	***$315.45***	**100.00%**

### Replication (Scale-up) Costs Using Four Different Implementation Scenarios

**Fig. 1:**
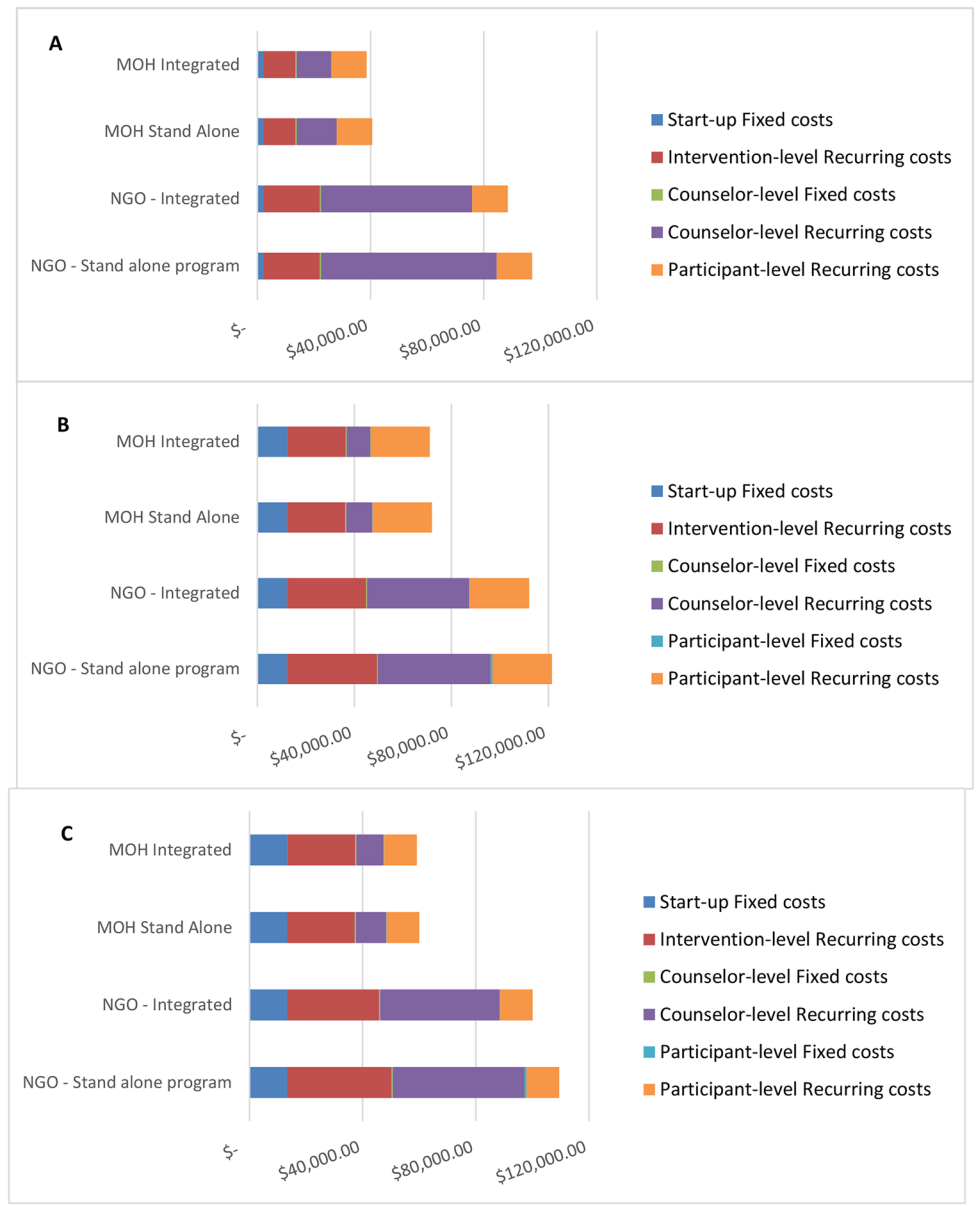
**Costs of Replication of Live Counselor, SMS and IVR Interventions using Four Hypothetical Implementation Models (N = 1000)** A. Live Counselor Delivered Booster-based Intervention B. SMS Delivered Booster-based Intervention C. IVR Delivered Booster-based Intervention

In Fig. 1, we illustrate the estimated cost of scale-up of replication of each intervention to 1000 participants using the four different scale-up scenarios described above, for each of the intervention booster delivery methods (Fig. 1a, 1b, and 1c). Similar to our observed results, we estimate that scale up to 1000 participants will be least expensive for the live call-delivered booster intervention, and most expensive for the SMS-delivered booster intervention. In addition, for each intervention, scale up would be least expensive if implemented by the MOH using an integrated model, followed by the MOH using a stand-alone model, and significantly more expensive if implemented by an NGO using an integrated or stand-alone model.

Variation in the distribution of types of costs across models was similar for each intervention. All four implementation scenarios have similar start-up and participant-level costs. The NGO scenarios have higher intervention-level recurring costs (i.e., program oversight, staff salaries and benefits, connectivity and recurring office expenses) compared to the MOH scenarios. Counselor-level costs (i.e., counselor salaries and benefits, as well as office furniture and supplies for counselors) differed across all scenarios and were higher for NGO scenarios compared with MOH scenarios and higher for stand-alone scenarios compared to integrated scenarios.

### Economy of Scale

**Fig. 2:**
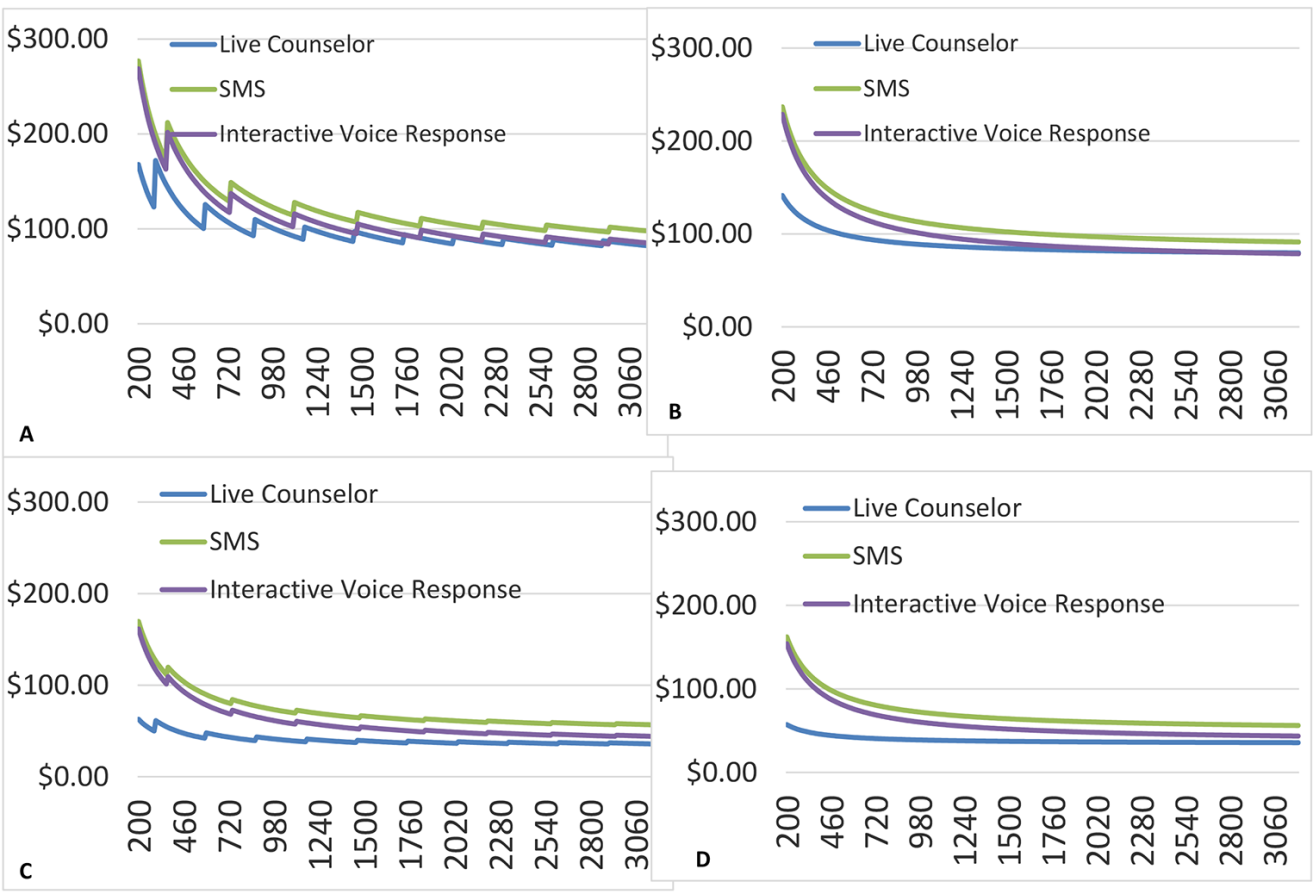
**Economy of Scale for Four Hypothetical Implementation Scenarios** A. Free-standing program, NGO counselors B. Integrated program, NGO counsellors C. Free-standing program, MOH counsellors D. Integrated program, MOH counsellors

In Fig. 2, we illustrate the estimated per participant replication cost for each intervention, by the four different implementation scenarios (2a through 2d). The stand-alone scenarios are characterized by a jagged curve due to increased costs when it is necessary to hire an additional counselor for each intervention as opposed to integrated scenarios that allow counselors to spread their time across other projects as appropriate. For each scenario and intervention, the per participant cost declines rapidly as the number of participants increases, and levels off above 1000 participants. In each scenario, the live call-delivered booster intervention is substantially less expensive than other interventions when the number of participants is small, but is similar to the IVR-delivered booster intervention in cost per participant when the number of participants is large (~ 1500). This is due to the relatively smaller start-up costs for the live call boosters compared with the IVR boosters. In contrast, the SMS and IVR booster-based interventions have similar costs when the number of participants is relatively small, but the SMS intervention is substantially more expensive than the IVR booster-based intervention when the number of participants is large. This difference is due to the similar start-up costs but larger patient-level costs (primarily for phone credit) for the SMS booster-based intervention compared to the IVR booster-based intervention.

## Discussion

In our implementation of three different modes of booster session delivery within a counseling-based alcohol intervention for PWH in Uganda, we found that the intervention including twice weekly booster sessions using automated technology systems cost 2.5-3 times more per participant than that of the intervention using phone calls from a live counselor every 3 weeks. This result was consistent over four different implementation scenarios for programs with 1000 participants. The differences in costs between the intervention delivery types were driven overall by significant differences between the technology and non-technology (live call) arms for capital and training costs. For example, there were several initial investments for locally developed software. In addition, associated recurring costs (i.e., recurring connectivity and technology support costs) were increased for the technology boosters compared to the live call arms. For the live call booster-based intervention arm, the observed costs per participant were higher than the costs reported for a very similar brief intervention tested in a study in Vietnam. However, the differences were due to costs used for counselor salaries; sensitivity analysis using MOH counselor salaries showed estimated costs that were quite similar to those observed in Vietnam ($38.60-$40.60 for hypothetical programs implemented for 1000 persons at MOH facilities, compared to $39 reported for the study in Vietnam) [18].

As technology use grows, however, these costs may decline over time. Based on these costs, for technology-based interventions (either SMS or IVR) to be considered cost-effective, the efficacy of an intervention (i.e., decreased drinking and/or other health improvements) using these technologies will need to be significantly greater for IVR- or SMS- based booster session delivery compared to an intervention that relies on live calls from counsellors for booster delivery if the program includes fewer than 1000 participants. It is notable that when participants in the SOC arm were offered the intervention after 9 months, the vast majority (87%) chose the live call option over the two technology-based options, suggesting the live call option was preferable in addition to being lowest per participant cost.

### Limitations

We were unable to provide cost-effectiveness because we did not find statistically significant effects in the main trial. The lack of efficacy in the trial may be because we included all persons with unhealthy alcohol use due to the dearth of mental health professionals in sub-Saharan Africa [27] and the intervention may not have been sufficient to help those with the highest levels of alcohol use. However, despite our inability to conduct cost-effectiveness, our findings illustrate the relative costs of live versus automated components and are instructive for future intervention development and implementation.

In addition, this analysis did not address key utilization issues such as the ease of use of the different booster delivery methods or participant considerations, such as privacy of receiving live calls as compared to receiving SMS or IVR messages that required written or push-button responses. A further limitation of this work is that the intervention delivery occurred within the context of a research study that scaled up three different interventions. Thus, our personnel and infrastructure costs were higher than for a typical HIV clinic, but we also accrued savings by being able to share resources across the differing interventions. Lastly, we chose the frequency of the booster sessions based on prior literature and based on what we considered most likely in future interventions; as such, the increased costs of the technology-based boosters compared to the live calls were in part driven by the high frequency of the technology-based booster sessions.

## Conclusion

While technology-based interventions have the potential to deliver more frequent participant contact, their start-up and maintenance costs are likely to exceed to costs of phone calls made by counselors in LMICs, with costs only beginning to equalize at large scale. These results are likely to be relevant for other counseling-based interventions, for example, smoking cessation or HIV risk reduction interventions, in which technology-based interventions are being considered. While technology-based intervention components may be appealing in LMICs because of their scalability, their costs are only likely to become equivalent to lower-technology solutions like counselor-delivered phone calls at very large scale.

## Data Availability

Data are available on request

## Code Availability

Software are available on request.
